# Integrative bioinformatics analysis identifies APOE as a candidate link between lipid dysregulation and macrophage activation in inborn errors of metabolism

**DOI:** 10.3389/fphar.2026.1880997

**Published:** 2026-07-13

**Authors:** Rahmat Dani Satria, Riswan Hadi Kusuma, Nazhipah Isnani, Usi Sukorini, Tri Ratnaningsih, Elizabeth Henny Herningtyas, Umi S. Intansari, Windarwati, Nur Imma Fatimah Harahap, Primadhy Harris Syaifullah, Kharisa Rasikhatul Hikmatul, Litia Lucretia Bodiwan, Richardus Wisnandito Prabasaktya, Nicholas Matthew Santoso, Muhammad Arkaan Putra Aprilia Darmawan, Eko Mugiyanto, Bambang Ardianto, Lalu Muhammad Irham, Chiou-Feng Lin, Chia-Ling Chen, Wirawan Adikusuma

**Affiliations:** 1 Clinical Pathology and Laboratory Medicine, Faculty of Medicine, Public Health and Nursing, Universitas Gadjah Mada, Yogyakarta, Indonesia; 2 Integrated Clinical Laboratory Installation, Universitas Gadjah Mada Academic Hospital, Yogyakarta, Indonesia; 3 Department of Pharmacy, Faculty of Mathematics and Natural Sciences, Lambung Mangkurat University, Banjarbaru, Indonesia; 4 Integrated Laboratory Installation, Dr. Sardjito General Hospital, Yogyakarta, Indonesia; 5 Department of Pharmacy, Faculty of Health Science, University of Muhammadiyah Pekajangan Pekalongan, Pekalongan Regency, Central Java, Indonesia; 6 Department of Child Health, Faculty of Medicine, Public Health, and Nursing, Universitas Gadjah Mada, Yogyakarta, Indonesia; 7 Faculty of Pharmacy, Universitas Ahmad Dahlan, Yogyakarta, Indonesia; 8 Graduate Institute of Medical Sciences, College of Medicine, Taipei Medical University, Taipei, Taiwan; 9 Department of Microbiology and Immunology, College of Medicine, Taipei Medical University, Taipei, Taiwan; 10 School of Respiratory Therapy, College of Medicine, Taipei Medical University, Taipei, Taiwan; 11 TMU Research Center of Thoracic Medicine, Taipei Medical University, Taipei, Taiwan; 12 Pulmonary Research Center, Wan Fang Hospital, Taipei Medical University, Taipei, Taiwan; 13 Research Center for Computing, Research Organization for Electronics and Informatics, National Research and Innovation Agency (BRIN), Cibinong, Indonesia

**Keywords:** APOE, bioinformatics, biomarkers, inborn errors of metabolism, macrophage activation syndrome

## Abstract

**Background:**

Inborn errors of metabolism (IEM) are a heterogeneous group of genetic disorders characterized by metabolic dysregulation and high mortality. Despite extensive genetic discoveries, the molecular mechanisms underlying severe disease progression remain incompletely understood. Increasing evidence suggests that host metabolic states, particularly lipid dysregulation, can profoundly influence immune cell function and may contribute to impaired responses against intracellular pathogens.

**Methods:**

We integrated genomic data from four high-confidence databases (GWAS Catalog, CTD, Open Targets, and ClinVar) and identified 1,288 IEM-associated genes. Disease ontology enrichment, multi-parametric functional annotation, and cell-type enrichment analyses were performed using Open-XGR, WebGestalt, and Tabula Sapiens. Bioinformatics findings were interpreted alongside clinical and cytomorphological observations from a pair of one-year-old twins with suspected IEM and macrophage activation syndrome. No patient-specific genetic or molecular validation of APOE was available.

**Results:**

Functional annotation identified APOE as the highest-scoring gene across seven biological categories, with enrichment in lipid metabolism- and macrophage-related pathways. Cell-type enrichment demonstrated an overrepresentation of IEM-associated genes in macrophage-related reference signatures, including datasets annotated as M2 macrophages. APOE was the only gene consistently represented across macrophage, M2 macrophage, protein–lipid complex, and vasculature-associated macrophage categories. Clinical evaluation of the twin cases showed persistent hyperbilirubinemia, elevated transaminases, cholestasis, and macrophage activation. Bone marrow and cerebrospinal fluid cytology demonstrated lipid-laden monocyte/macrophage morphology. These clinical observations are concordant with lipid-handling abnormalities in macrophages but do not establish an APOE-dependent mechanism or macrophage polarization state.

**Conclusion:**

This study identifies APOE as a highly prioritized candidate gene associated with lipid metabolism pathways and macrophage-related signatures in IEM. These findings support the hypothesis that APOE-associated lipid dysregulation may contribute to macrophage dysfunction and warrant further experimental validation.

## 1 Introduction

Inborn errors of metabolism (IEM), also referred to as inherited metabolic disorders (IMD), are caused by disruptions in biochemical pathways resulting from deficiencies in enzymes, cofactors, or transporters (Mordaunt et al., 2020; Balakrishnan, 2021). These disorders constitute a complex and heterogeneous group of rare diseases characterized by diverse clinical manifestations and significant morbidity and mortality (Mordaunt et al., 2020; Balakrishnan, 2021; Den Hollander et al., 2025). According to the International Classification of Inherited Metabolic Disorders (ICIMD), 1,546 IEMs have been identified to date, and this number continues to increase with advancements in analytical technologies that elucidate the molecular and metabolic basis of previously undefined conditions (Den Hollander et al., 2025). Although each disorder is individually rare, their combined incidence is considerable, estimated to occur in approximately one in every 800 to 2,000 live births (Balakrishnan, 2021; Den Hollander et al., 2025).

IEMs result from genetic variations that impair the function of proteins essential for cellular metabolism, leading to either a deficiency in energy production or the accumulation of pathological macromolecules (Mordaunt et al., 2020; Barbosa-Gouveia et al., 2021). Substrates that are normally metabolized may accumulate, causing disease-specific clinical manifestations (Mordaunt et al., 2020; Barbosa-Gouveia et al., 2021). The clinical presentation of IEMs varies widely, even among individuals with the same disorder. Some conditions display distinctive features that facilitate diagnosis, while others manifest with nonspecific or subtle symptoms and are often identified incidentally through laboratory testing (Mordaunt et al., 2020; Balakrishnan, 2021; Barbosa-Gouveia et al., 2021). A high index of clinical suspicion is essential for accurate diagnosis, as many IEMs are amenable to treatment, and early detection can significantly reduce mortality (Mordaunt et al., 2020; Balakrishnan, 2021).

Recent advances in bioinformatics have facilitated the integration of genomic databases with functional genomics resources, providing deeper insights into disease mechanisms and enabling the identification of factors contributing to mortality in IEM (Mordaunt et al., 2020; Barbosa-Gouveia et al., 2021; Zandl-Lang, 2024). In this study, we combined population-level bioinformatics analyses with descriptive clinical and cytomorphological observations from twin patients with suspected IEM. The clinical cases were included to provide phenotypic context for the bioinformatics findings; however, patient-specific genetic or molecular evidence linking APOE to the observed phenotype was unavailable. Therefore, the aim of this study is to identify distinctive molecular and phenotypic signatures of IEM that may serve as reliable indicators for accurate diagnosis and treatment planning.

## 2 Methods

### 2.1 Study design

**FIGURE 1:**
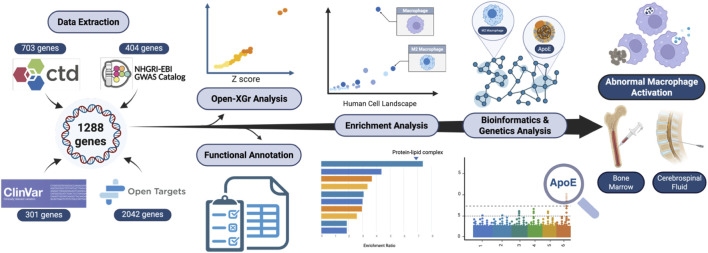
Comprehensive workflow of the bioinformatics and genetic analysis for inborn errors of metabolism (IEM). Genes associated with IEM were extracted from multiple databases resulting in a total of 1,288 genes. Disease ontology enrichment analysis using the Open-XGR platform confirmed that these genes were highly enriched in IEM-related pathways. Functional annotation and enrichment analyses were then performed using cell-related databases such as the Human Cell Landscape, Cellular Component, and Tabula Sapiens. Integration of bioinformatics findings and descriptive clinical observations prioritized APOE as a candidate gene associated with lipid metabolism and macrophage-related signatures in IEM.

The analytical workflow in this study is shown in Figure 1. Genes linked to IEM were first retrieved from four high-confidence repositories: the NHGRI-EBI GWAS Catalog, the Comparative Toxicogenomics Database (CTD), the Open Targets Platform, and ClinVar. After merging datasets and removing duplicates, 1,288 unique genes were retained. To confirm that this gene set reflected IEM-related biology, disease ontology enrichment was conducted via the Open-XGR platform. Gene sets were then subjected to functional annotation. Subsequent bioinformatics analyses were integrated: (1) cell-type and tissue-specific expression profiles from the Human Cell Landscape, Cellular Component, and Tabula Sapiens; and (2) significantly expressed genes highlighted in the Manhattan plot. Joint interpretation of the bioinformatics outputs identified APOE as a candidate associated with lipid metabolism and macrophage-related reference signatures. The clinical and cytomorphological findings were evaluated as phenotypic context for macrophage activation and lipid accumulation but were not considered evidence of APOE causality or of a defined M2 polarization state in the patients.

### 2.2 Disease ontology enrichment

Disease ontology enrichment was conducted using Open-XGR (Bao et al., 2023; accessed 20 May 2025) to evaluate the biological validity of the integrated gene list. The analysis used curated disease ontology annotations and hypergeometric testing with multiple-testing correction. Overrepresentation of inherited metabolic disorder terms and related metabolic phenotypes supported the relevance of the gene set for subsequent functional scoring and mechanistic evaluation.

### 2.3 Data extraction of IEM-Associated genes

An extensive data-mining approach was undertaken to systematically identify and consolidate genes implicated in the pathogenesis of IEMs. Four well-established and high-confidence bioinformatics repositories, each recognized for their comprehensive coverage of genetic and disease-associated information, were accessed on 20 May 2025. These included the GWAS Catalog (https://www.ebi.ac.uk/gwas/home) (Sollis et al., 2023), the Comparative Toxicogenomics Database (CTD; https://ctdbase.org/) (Davis et al., 2023), the Open Targets Platform (https://www.targetvalidation.org/) (Ochoa et al., 2023), and ClinVar (https://www.ncbi.nlm.nih.gov/clinvar/) (Landrum et al., 2020). For each database, searches were performed using the standardized keyword “inborn errors of metabolism,” ensuring consistent retrieval across data sources. The GWAS Catalog was utilized to extract genes significantly associated with IEMs based on genome-wide association studies, while the CTD and Open Targets Platform were leveraged to integrate evidence from curated toxicogenomic relationships and target-disease associations, respectively. ClinVar was further employed to obtain clinically validated variants linked to metabolic disorders. To enhance data reliability, a relevance filtering procedure was implemented within the Open Targets Platform, whereby only genes with an association score greater than 0.3 were retained, reflecting moderate to strong confidence in disease relevance (Adikusuma et al., 2023; Adikusuma et al., 2025). After extraction, all gene lists from the four databases were merged, and duplicate entries were removed to generate a non-redundant, high-confidence gene compendium comprising 1,288 genes. This curated dataset serves as a foundational and integrative resource for subsequent analyses.

### 2.4 Functional annotation scoring of IEM genes

To prioritize genes with potential biological relevance to IEM, a multi-domain functional annotation and scoring framework was applied. Seven complementary annotation domains were integrated to capture functional, cellular, phenotypic, and disease-related evidence: (1) Gene Ontology Biological Process, representing the biological programs and cellular processes in which a gene participates; (2) Gene Ontology Cellular Component, describing its subcellular or extracellular localization; (3) Gene Ontology Molecular Function, reflecting its biochemical, catalytic, or binding activities; (4) DisGeNET, providing curated and literature-supported evidence of gene–disease associations; (5) the Human Cell Landscape, providing tissue- and cell-type expression context for candidate genes; (6) the Human Phenotype Ontology, linking genes to clinically defined phenotypic abnormalities; and (7) the Kyoto Encyclopedia of Genes and Genomes, providing pathway-level information on gene involvement in metabolic, signaling, and disease-associated networks.

All enrichment analyses were performed using WebGestalt 2024, with statistical significance defined as a false discovery rate-adjusted q value <0.05 (Elizarraras et al., 2024). Each gene received a cumulative annotation score based on the number of criteria fulfilled. For a given annotation domain, a gene was assigned a score of 1 if it was represented in at least one significantly enriched term within that domain and a score of 0 if it was not represented. A cumulative score of ≥5 was selected as an operational threshold to identify genes supported by at least five of the seven domains. Genes meeting this threshold were designated high-priority IEM-associated candidate genes. This cutoff was intended for exploratory candidate prioritization and was not derived from a validated statistical classifier or predictive probability. Higher scores indicate broader cross-domain annotation support and should not be interpreted as direct evidence of mechanistic involvement, disease causality, or clinical risk.

### 2.5 Clinical case assessment

Clinical data from one-year-old twins with clinically suspected inborn errors of metabolism (IEM) were reviewed between July and October 2025. Comprehensive molecular characterization, including identification of causative genetic variant(s), was not available at the time of analysis. Likewise, definitive biochemical characterization of the underlying metabolic pathway defect could not be established. Patient-specific APOE genotyping, targeted sequencing, whole-exome sequencing, APOE transcript or protein measurements, and APOE immunostaining were not performed. Therefore, the clinical assessment was descriptive and was not designed to establish an APOE-associated diagnosis or a direct relationship between APOE and the observed macrophage phenotype. Laboratory profiles included complete blood counts, liver function tests, lipid panels, and serum biochemistry. Peripheral blood hematology profiles were analyzed using the Sysmex XN-1000 analyzer, while serum biochemical parameters were measured using the Cobas Pro 1 system. Cytospin cerebrospinal fluid (CSF) analysis was performed to identify monocyte infiltration within the central nervous system. Bone marrow analysis was conducted by a hematology specialist. A differential count was performed on 500 cells, and inter-observer agreement was assessed using Cohen’s kappa coefficient between two independent experts. Available metabolic characterization was limited to routine biochemical and lipid profiles. Disease-specific enzyme assays, comprehensive metabolomic or lipidomic profiling, and confirmatory biochemical testing for a defined IEM subtype were not available.

### 2.6 Statistical analysis

All gene extractions were performed using the NHGRI-EBI GWAS Catalog (https://www.ebi.ac.uk/gwas/home), the Comparative Toxicogenomics Database (CTD; https://ctdbase.org/), the Open Targets Platform (https://www.targetvalidation.org/), and ClinVar (https://www.ncbi.nlm.nih.gov/clinvar/). The Manhattan plot was generated using RStudio version 2024.09.0 + 375 (RStudio, Boston, MA, USA). Functional annotation was performed using the WEB-based GEne SeT AnaLysis Toolkit (WebGestalt; https://www.webgestalt.org), covering seven different annotation categories. Enrichment analyses were conducted using two databases, WebGestalt and Open-XGR (http://www.openxgr.com). Gene network analysis was carried out through Enrichr (https://maayanlab.cloud/enrichr-kg). All database searches and bioinformatics analyses were performed on 20 May 2025.

## 3 Results

### 3.1 Disease ontology enrichment analysis of IEM-Associated genes

**FIGURE 2:**
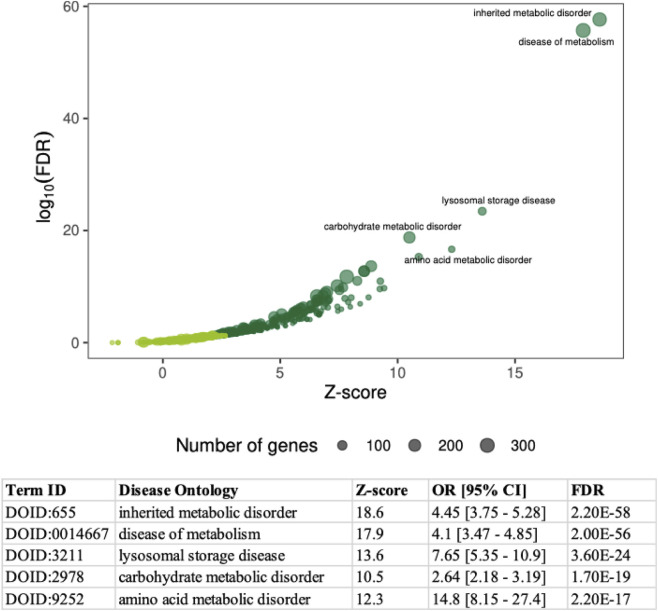
Disease ontology enrichment analysis of genes associated with inborn errors of metabolism (IEMs). Bubble plot showing the top enriched disease ontology terms identified by Open-XGR from 1,288 genes extracted from four databases. Bubble size indicates the number of genes per term. The strongest enrichment was observed for inherited metabolic disorder, confirming that the integrated gene set is highly associated with IEM.

A total of 1,288 candidate genes were identified through extraction from the GWAS Catalog, CTD, Open Targets Platform, and ClinVar (Supplementary Table 1). To confirm that this integrated dataset was truly related to inborn errors of metabolism (IEMs), disease ontology enrichment analysis was performed using Open-XGR (Figure 2). This step was necessary because gene lists aggregated from heterogeneous sources may include genes associated with unrelated conditions.

The analysis demonstrated strong enrichment for multiple categories of metabolic disorders. The highest enrichment was seen for inherited metabolic disorder, with a Z-score of 18.6, an odds ratio (OR) of 4.45, and an FDR of 2.20 × 10^−58^. Similar enrichment was observed for disease of metabolism terms (Z-score 17.9; OR 4.1; FDR 2.00 × 10^−56^) and specific subtypes, including lysosomal storage disease, carbohydrate metabolic disorder, and amino acid metabolic disorder. These results confirm that the integrated gene set is strongly aligned with IEM-related pathways.

### 3.2 Functional annotation prioritization reveals key genes implicated in IEM

**FIGURE 3:**
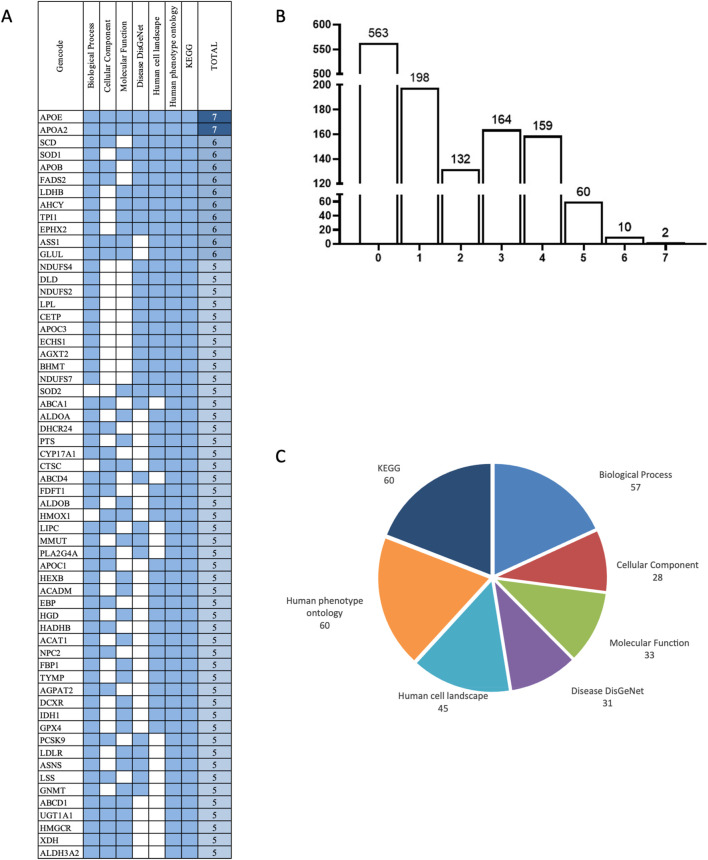
Multi-parametric functional annotation and scoring of 1288 IEM-associated genes **(A)** Table showing the integration of seven annotation features, including biological process, cellular component, molecular function, DisGeNET, Human Cell Landscape, Human Phenotype Ontology, and KEGG pathway memberships. Genes with higher cumulative scores indicate greater biological relevance **(B)** Distribution of genes by total annotation score, with 58 genes achieving scores ≥5 **(C)** Pie chart summarizing the number of enriched terms across the seven annotation categories. APOE and APOA2 obtained the highest composite scores, indicating representation across all seven annotation domains.

A multi-parametric functional annotation and scoring framework was applied to prioritize genes with potential biological relevance to IEM (Supplementary Table 2; Figure 3). Seven annotation categories were incorporated: (1) essential biological processes; (2) subcellular localization; (3) molecular function; (4) DisGeNET disease associations; (5) Human Cell Landscape expression specificity; (6) Human Phenotype Ontology (HPO) annotations; and (7) KEGG pathway memberships. Annotation analyses were conducted using WebGestalt 2024. Each gene received a cumulative score based on the number of fulfilled criteria. Genes with composite scores ≥5 were designated as high-priority IEM-associated candidate genes, reflecting strong functional convergence and mechanistic relevance.

The integration results identified APOE, followed by APOA2, as the top-scoring genes, each fulfilling all seven annotation categories (Figure 3A). This prioritizes APOE as a pleiotropic candidate represented across multiple metabolic and regulatory annotation domains relevant to IEM. Score distribution analysis showed a right-skewed pattern (Figure 3B). Most genes (n = 562) scored <1, indicating limited annotation support, while only 58 genes achieved scores ≥5 and were designated high-priority IEM-associated candidate genes. This stratification highlights a focused subset of biologically relevant candidate genes. Across the seven annotation domains, KEGG pathways and HPO contained the highest number of enriched terms (n = 60), followed by biological processes (n = 57) and Human Cell Landscape (n = 45) (Figure 3C). The broad functional coverage emphasizes the multifaceted involvement of IEM-related genes across molecular, cellular, and phenotypic levels.

### 3.3 Cell-type enrichment analysis of the 1,288 IEM-Associated genes

Several studies have discussed the relationship between macrophages and inborn errors of metabolism (Escolà-Gil et al., 2021; Ricci et al., 2024; Ferreira et al., 2025). For that reason, we want to determine which cell types these 1,288 genes are involved in forming. This is to investigate what defects occur in cells in inborn errors of metabolism. It may potentially be useful as a diagnostic marker for IEM.

**FIGURE 4:**
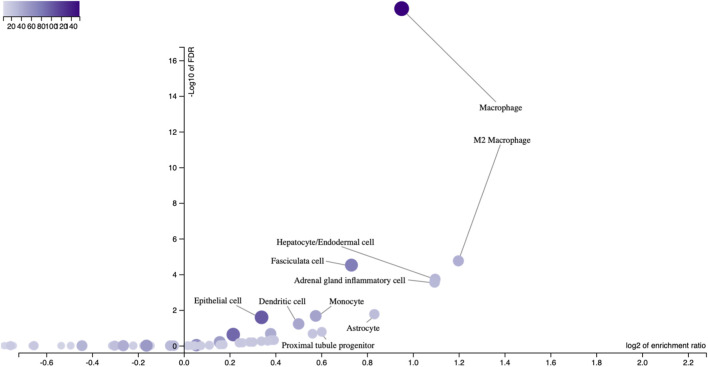
Cell-type enrichment analysis of the 1,288 IEM-associated genes. The analysis identified significant overlap between the IEM-associated gene set and macrophage-related reference expression signatures, including a dataset annotated as M2 macrophages. These findings do not establish macrophage abundance, functional activation, or polarization in patient samples. This figure was generated using WebGestalt (https://www.webgestalt.org).

Our cell-type enrichment analysis indicates that the 1,288 IEM-associated genes are predominantly linked to macrophage-related signatures, including reference datasets annotated as M2 macrophages (Figure 4). However, these enrichment results should not be interpreted as direct evidence of macrophage polarization states in patient samples. Since M2 macrophages rely on oxidative phosphorylation and lipid metabolic pathways, defects in these processes may impair macrophage polarization and function in IEM (Remmerie and Scott, 2018; van Eijk and Aerts, 2021; Vassiliou and Farias-Pereira, 2023; Zandl-Lang, 2024; Ferreira et al., 2025). This finding aligns with recent evidence showing that metabolic gene abnormalities can disrupt immune cell programming (van Eijk and Aerts, 2021; Zandl-Lang, 2024; Ferreira et al., 2025; Mohit et al., 2025). Therefore, macrophage-related pathways may be relevant in a subset of IEMs characterized by lipid accumulation or immune dysregulation and could serve as a promising basis for the development of diagnostic marker. These findings indicate enrichment in macrophage-related reference signatures, including datasets annotated as M2 macrophages, suggesting that metabolic dysregulation may influence immune cell states that are also critical in host responses to intracellular pathogens.

### 3.4 APOE is prioritized at the intersection of macrophage-related signatures and protein–lipid complexes

**FIGURE 5:**
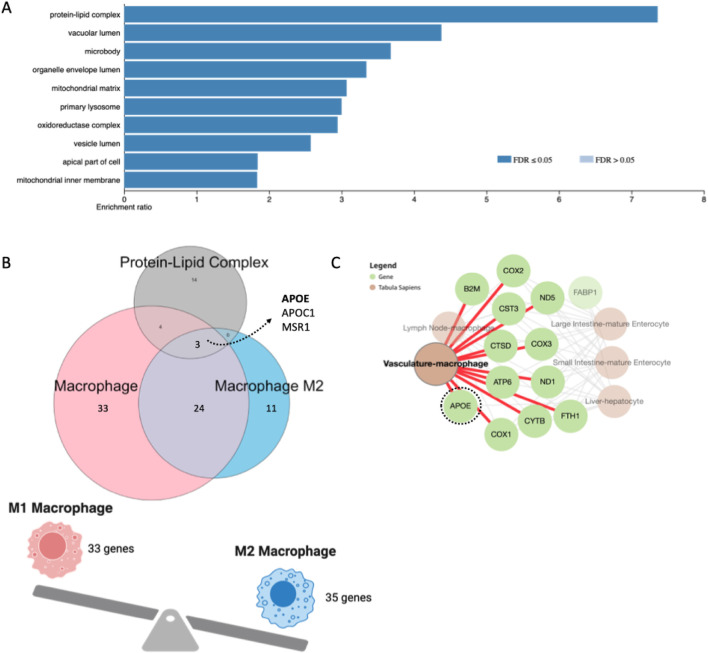
Cellular component enrichment and macrophage subtype-associated gene overlap in IEMs **(A)** Cellular component enrichment analysis of the 1,288 IEM-associated genes showing highest significant enrichment in the protein–lipid complex **(B)** Venn diagram showing the overlap between genes associated with macrophages, M2 macrophages, and the protein–lipid complex. A total of 35 genes correspond to M2 macrophage differentiation and 33 to M1 macrophage function. The larger number of genes involved in M2 macrophage reference signatures suggests that M2-associated transcriptional signatures were slightly more represented than M1-associated signatures within the enrichment dataset. Three genes (APOE, APOC1, and MSR1) are shared across macrophage subsets and the protein–lipid complex, indicating an overlap between macrophage-associated reference signatures and protein–lipid complex annotations **(C)** Cell-type enrichment validation using the Tabula Sapiens single-cell atlas. Of the 1,288 genes queried, only 12 genes were specifically associated with the vasculature–macrophage population. APOE appears as the only consistently overlapping gene across macrophage subsets and protein–lipid complex categories, supporting its potential relevance to IEM-related macrophage biology.

To further characterize the cellular features associated with IEM genes, we examined cellular component enrichment. The most significant enrichment was observed for the protein–lipid complex category (Figure 5A), consistent with the known involvement of lipid and protein metabolism in IEMs (Mordaunt et al., 2020; Balakrishnan, 2021; Zandl-Lang, 2024; Den Hollander et al., 2025).

Next, we assessed overlapping genes associated with macrophage and M2 macrophage reference signatures. Thirty-five genes were linked to M2 macrophage differentiation, and 33 to M1 macrophage differentiation. The enrichment of genes associated with M2 macrophage reference signatures suggests that lipid-handling macrophage programs may be relevant to IEM pathophysiology. When these were intersected with protein–lipid complex genes, APOE, APOC1, and MSR1 emerged as shared genes (Supplementary Table 3, Figure 5B).

To assess cross-resource consistency, we performed an independent cell-type enrichment analysis using the Tabula Sapiens single-cell atlas. Of the 1,288 genes, only 12 mapped specifically to vasculature-associated macrophages (Figure 5C). APOE was the only gene consistently overlapping across macrophages, M2 macrophages, protein–lipid complex and vasculature-macrophages. This cross-analysis overlap further supports the prioritization of APOE as a candidate associated with macrophage-related and lipid-handling pathways. Consistent with this, APOE also received the highest functional annotation score (Figure 3A).

**FIGURE 6:**
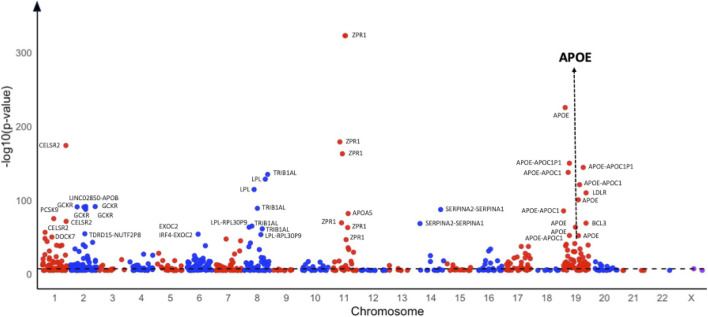
Manhattan plot of genome-wide association analysis for IEM. The horizontal dashed line indicates the genome-wide significance threshold (P < 5 × 10^−8^). Variants surpassing this threshold are considered to have significant associations with IEM. Notably, two genomic loci demonstrated the strongest signals: ZPR1 located on chromosome 11 and APOE located on chromosome 19. However, only APOE demonstrated consistent relevance across all analytical layers in this study, providing an additional, independent layer of association supporting its prioritization for further investigation.

To further assess APOE’s genetic significance, we examined IEM-related GWAS summary statistics (Supplementary Table 4). Chromosomal mapping revealed two notable loci: ZPR1 (chromosome 11) and APOE (chromosome 19) (Figure 6). Although ZPR1 is implicated in a rare developmental disorder (Ito et al., 2018; Quintana et al., 2025), it received no annotation score in our analysis and lacks established mechanistic links to IEM. In contrast, APOE demonstrated consistent relevance across all analytical layers in this study, providing an additional, independent layer of association supporting its prioritization for further investigation.

Based on the multiple analytical approaches performed in this study, APOE emerged as the highest-priority candidate gene associated with protein–lipid metabolism pathways and macrophage-related signatures that may contribute to macrophage lipid-handling dysfunction. The importance of APOE is further supported by its high level of significance in the Manhattan plot derived from the GWAS dataset. This finding is consistent with previous studies that have similarly reported APOE’s involvement in key IEM-related processes. These findings nominate APOE for evaluation in future studies examining its potential utility as a molecular or pathway-level marker in defined IEM subgroups. Given the central role of macrophages as host cells for intracellular pathogens, the identification of APOE as a candidate gene associated with lipid-associated macrophage pathways may have broader implications for host–pathogen interactions. It should be noted that the GWAS-derived associations and functional annotations were obtained from public datasets and were not generated from the twin patients. Accordingly, the prioritization of APOE cannot be interpreted as evidence that either patient carried an APOE variant or had altered APOE expression.

### 3.5 Twin case studies

The twin cases are presented as descriptive clinical observations and were not genetically linked to APOE. Although their clinical and cytomorphological findings were compatible with macrophage activation and abnormal intracellular substrate accumulation, the underlying IEM subtype remained unconfirmed. In this case, a pair of one-year-old twins presented to the hospital with similar clinical signs and symptoms. The clinical presentation was highly suggestive of an underlying IEM; however, definitive molecular classification was unavailable. At 11 days of age, both infants exhibited elevated total and direct bilirubin levels that did not normalize with phototherapy. Additionally, AST and ALT levels were markedly elevated above the reference ranges during this neonatal period.

By 1 year of age, both twin patients presented in a deteriorating condition requiring intensive care. At this time, the biochemical abnormalities persisted, with AST and ALT levels remaining several-fold higher than normal, accompanied by reduced albumin levels. Both children also continued to exhibit elevated bilirubin concentrations. Bilirubin is a strong clinical indicator of abnormalities associated with inborn errors of metabolism (Ozen et al., 2014; Heubi et al., 2018), as physiologic hyperbilirubinemia typically resolves within 2–3 weeks after birth; however, in these twins, bilirubin levels remained persistently elevated even at 1 year of age. This biochemical pattern reflects ongoing hepatocellular injury and provides strong evidence supporting an underlying inborn metabolic disorder.

In IEM, immune dysregulation occurs within hepatic macrophages, leading to the activation of inflammatory signaling pathways (Ozen et al., 2014; Tarasenko and McGuire, 2017; Roohani and Tacke, 2021). This process disrupts the excretion of conjugated (direct) bilirubin from the liver into the gastrointestinal tract due to inflammation and fibrosis along the biliary excretory pathways caused by aberrant macrophage activity (Ozen et al., 2014; Li et al., 2017; Chen and Zhang, 2023; Horn and Tacke, 2024). As a result, secondary cholestasis develops, which is reflected by elevated GGT levels in both infants. The co-occurrence of cholestasis and hyperbilirubinemia is recognized as one of the hallmarks of secondary hemophagocytic lymphohistiocytosis (HLH) or macrophage activation syndrome (MAS) (Ozen et al., 2014).

**FIGURE 7:**
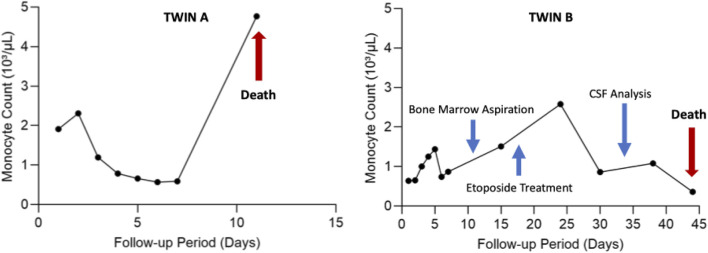
Time-Course of Monocyte Counts and Clinical Interventions in Twin Infants with IEM. The graphs illustrate the temporal changes in absolute monocyte counts (×10^3^/μL) during the intensive care follow-up period in Twin A (left) and Twin B (right). Twin A demonstrated a progressive rise in monocyte count, peaking at 4.77 × 10^3^/μL on day 10, followed by rapid clinical deterioration and death before treatment could be initiated. Twin B underwent bone marrow aspiration on day 12, confirming macrophage activation syndrome. Etoposide therapy was initiated shortly thereafter; although the monocyte count increased to 2.58 × 10^3^/μL on day 24, it subsequently showed a gradual decline. Subsequent cerebrospinal fluid (CSF) analysis revealed monocyte infiltration, preceding neurological deterioration and eventual death on day 44. Arrows indicate key clinical events: bone marrow aspiration, initiation of etoposide therapy, CSF analysis, and time of death.

**FIGURE 8:**
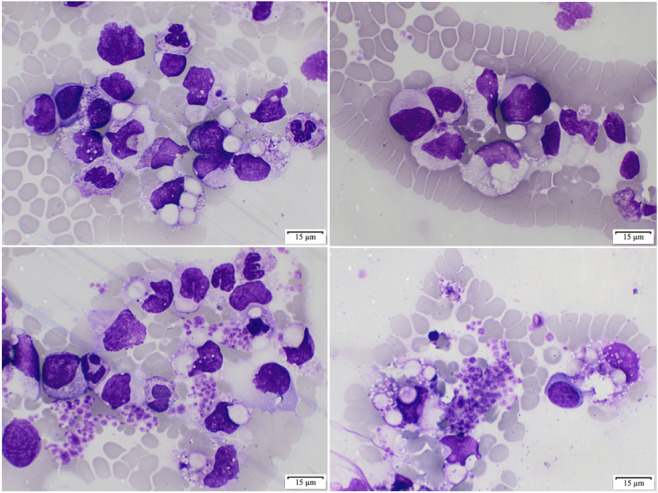
Bone marrow aspirate findings demonstrating excessive lipid-laden macrophages. Numerous enlarged macrophages with abundant, finely vacuolated cytoplasm are present, indicating intracellular lipid accumulation. These features are consistent with lipid-laden macrophages exhibiting prominent cytoplasmic vacuolization suggestive of intracellular lipid accumulation. All images were obtained from one of the twins, as a bone marrow procedure could not be performed in the other due to clinical deterioration.

On day 10 of the intensive care follow-up period, the condition of one of the twins (Twin A) deteriorated, accompanied by a marked rise in the absolute monocyte count, reaching 4,770 cells/μL. Consequently, on day 12, a bone marrow aspiration was performed in Twin B to establish a definitive diagnosis for suspected MAS (Figure 7). The evidence of MAS was supported by the presence of recurrent, fluctuating fever over several months, elevated serum triglyceride levels, and clear morphology of excessive macrophage activity in the bone marrow (Figure 8). This heightened macrophage activation was further reflected by an increased absolute monocyte count in the peripheral blood of both twins (Figure 7).

**FIGURE 9:**
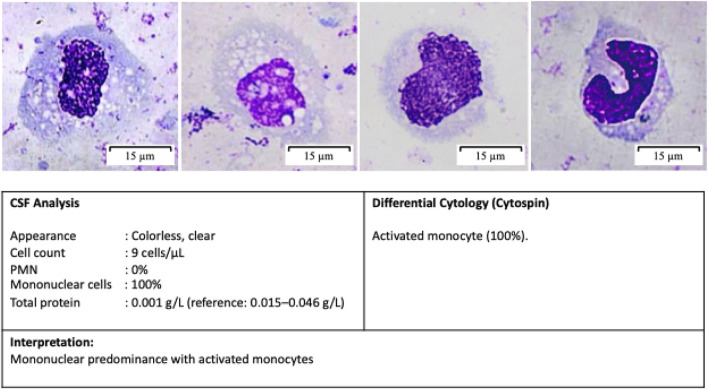
Cytological analysis of cerebrospinal fluid demonstrating infiltration of activated, lipid-laden monocytes/macrophages in Twin B. Cytospin preparation of cerebrospinal fluid (CSF) from Twin B showing abundant activated monocytes/macrophages with vacuolated cytoplasm consistent with lipid-laden macrophage morphology. These findings indicate mononuclear predominance with infiltration by activated, lipid-laden macrophages, representing central nervous system involvement secondary to macrophage activation syndrome (MAS) and correlating with the patient’s progressive neurological deterioration and refractory seizures.

Unfortunately, Twin A did not survive. The infant died shortly after the monocyte count peaked at 4,770 cells/μL and before treatment could be initiated, as the definitive diagnosis had not yet been established. Once the bone marrow aspirate confirmed macrophage activation syndrome (MAS), Twin B was treated with corticosteroids and etoposide. Although the monocyte count initially increased to 2,580 cells/μL on day 24 of follow-up, it subsequently declined with ongoing therapy. Twin B’s condition later deteriorated due to progressive neurological involvement, characterized by reduced consciousness and recurrent seizures. Cerebrospinal fluid (CSF) analysis demonstrated infiltration of activated monocytes (Figure 9). The patient ultimately died as a result of persistent, refractory seizures, representing a severe neurological complication secondary to macrophage infiltration into the CSF.

The increased presence of macrophages in the bone marrow aspirate indicates an intensified effort by the body to eliminate accumulating abnormal metabolic substrates. The enlarged vacuolated macrophages may reflect altered intracellular substrate handling; however, their lipid content, functional state, and polarization phenotype were not directly characterized (Remmerie and Scott, 2018; Wang et al., 2021; Schilperoort et al., 2023; Vassiliou and Farias-Pereira, 2023; Ferreira et al., 2025). Under physiological conditions, excessive intracellular lipid accumulation activates transcription factors such as PPARγ and LXR agonists, driving macrophages toward an adaptive M2 phenotype (Wang et al., 2021; Vassiliou and Farias-Pereira, 2023). However, in inborn errors of metabolism (IEM), excessive buildup of lipid substrates leads to macrophage lysosomal dysfunction, impairing lipid efflux and disrupting normal resolution pathways (van Eijk and Aerts, 2021; Koncz et al., 2023; Schilperoort et al., 2023; Yang et al., 2023). Consequently, macrophages may acquire a dysfunctional lipid-laden phenotype, potentially associated with impaired lysosomal processing and altered lipid handling. The presence of enlarged vacuolated macrophages in bone marrow aspirates (Figure 8) is consistent with intracellular lipid accumulation.

In Twin B, etoposide was initiated due to its well-established *in vivo* ability to suppress the excessively activated macrophages characteristic of HLH or MAS. A recent meta-analysis demonstrated that etoposide is effective in reducing cytokine storm activity and limiting the proliferation of hyperactivated inflammatory cells, particularly when administered in combination with corticosteroids (Gao et al., 2025). Despite its systemic efficacy, etoposide has poor penetration across the blood–brain barrier (Wei et al., 2021). Despite systemic treatment, subsequent CSF cytology demonstrated activated monocytes/macrophages. Limited central nervous system penetration of etoposide may have contributed to incomplete control of CNS inflammation; however, this possibility was not directly evaluated. This central nervous system involvement ultimately led to severe neurological complications, resulting in the infant’s death.

## 4 Discussion

Inborn Errors of Metabolism (IEM) arise from genetic variations that disrupt the function of proteins required for cellular metabolism, leading to impaired energy production or the accumulation of pathological macromolecules (Mordaunt et al., 2020; Balakrishnan, 2021). Most IEMs are caused by defects in a single gene encoding a specific enzyme within a metabolic pathway. When metabolism is blocked due to a dysfunctional enzyme, the system attempts to reach a new adaptive steady state that differs from normal physiological homeostasis. Tracer-lipidomics has demonstrated specific disturbances in lipid biosynthesis and remodeling pathways across various IEMs, including the accumulation of abnormal lipids and altered lipid flux (Zandl-Lang, 2024).

Although the enrichment analysis identified macrophage and M2 macrophage-associated signatures, these findings should not be interpreted as evidence of a fixed M2 polarization state. Macrophage activation exists along a dynamic continuum rather than a strict M1/M2 dichotomy. In the present study, the term “M2-like” refers only to bioinformatic enrichment of lipid-handling macrophage signatures and not to experimentally confirmed alternative macrophage polarization. The clinical syndrome observed in the twins is consistent with macrophage activation syndrome, a hyper-inflammatory macrophage-driven condition. The lipid-laden morphology in bone marrow and CSF is therefore interpreted as evidence of macrophage lipid-handling dysfunction rather than proof of anti-inflammatory M2 macrophage predominance.

**FIGURE 10:**
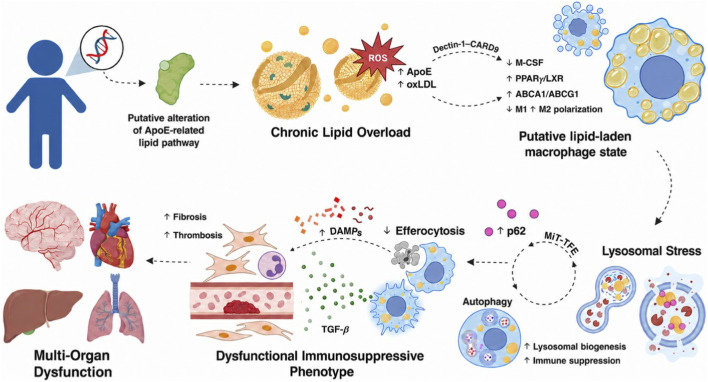
Hypothesis-generating model of the potential relationship among APOE-associated lipid pathways, macrophage lipid handling, and disease manifestations in IEM. The model integrates the bioinformatics findings of the present study with mechanisms proposed in previously published literature. APOE was prioritized at the intersection of lipid metabolism and macrophage-related reference signatures, whereas the clinical cases demonstrated macrophage activation and vacuolated macrophage morphology. The proposed relationships among altered APOE-related lipid handling, macrophage dysfunction, lysosomal stress, inflammation, fibrosis, thrombosis, and multi-organ involvement were not directly tested in the twin patients. The model should therefore be interpreted as a framework for future experimental investigation rather than an established causal pathway.

Based on the findings of this study, we propose a hypothesis-generating framework that may explain how APOE-associated lipid dysregulation could contribute to macrophage dysfunction and disease progression in IEM (Figure 10). Although the proposed model integrates findings from the current study with published literature, several downstream processes depicted in Figure 10, including impaired autophagy, ferroptosis, fibrosis, thrombosis, and intracellular pathogen persistence, were not directly evaluated. Therefore, these mechanisms should be regarded as speculative and hypothesis-generating rather than experimentally demonstrated within the present study. APOE is a major apolipoprotein that regulates lipid metabolism. It is expressed in multiple tissues, including the liver, brain, spleen, kidneys, gonads, adrenal glands, and macrophages. The APOE gene is located on chromosome 19q13.2 and encodes a 317-amino-acid apolipoprotein E precursor (Khalil et al., 2021). Under normal physiological conditions, APOE is incorporated into VLDL and chylomicrons to facilitate lipid clearance from the plasma and acts as a ligand for LDL receptors (LDLR) on hepatocytes. Macrophages also secrete APOE and take up APOE-containing lipoproteins to maintain lipid homeostasis in peripheral tissues. In individuals with APOE mutations or variants, the affinity of APOE for the LDL receptor is reduced, leading to impaired clearance of chylomicron and VLDL remnants by the liver. As a result, plasma lipoprotein levels—particularly total cholesterol and triglycerides—become elevated. Excessive lipid levels upregulate macrophage-activating surface markers (CD11b, CCR2, CD63) and stimulate macrophages to take up large quantities of lipids. Lipid-laden macrophages subsequently transform into foam cells, which accumulate within the circulation and tissues (Ferreira et al., 2025). This mechanism also occurs in several IEM conditions, such as APOE gene mutations, genetic defects affecting macrophage lysosomes, or disruptions in intracellular cholesterol transport proteins, all of which lead to the buildup of abnormal lipid substrates within macrophages (Remmerie and Scott, 2018; van Eijk and Aerts, 2021). Cholesterol accumulation in macrophages activates transcription factors including PPARγ, LXRs, and RXRs, which then increase the expression of ABCA1 and ABCG1, key regulators of cholesterol efflux and scavenger receptors. In addition to promoting cholesterol efflux transporters, these transcription factors also induce the expression of apolipoproteins such as APOE and APOC, which function as receptors for cholesterol (Remmerie and Scott, 2018; van Eijk and Aerts, 2021). However, during chronic lipid accumulation, defects in lipid efflux emerge and APOE becomes abnormally enriched on remnant particles, resulting in dysfunctional lipid remodelling (Remmerie and Scott, 2018).

The accumulation of cholesterol and oxidized lipids within macrophages induces ER stress, mitochondrial dysfunction, increased ROS, and impaired mitophagy, thereby altering the cell’s metabolic profile. Although oxidative stress is typically associated with inflammatory activation (M1), in conditions of chronic lipid overload such as IEM, lipid-derived ligands (e.g., oxidized low-density lipoproteins [ox-LDLs]) activate transcriptional receptors such as PPARγ/LXR agonists, causing macrophages to shift toward an M2-like phenotype focused on lipid handling and tissue repair (Remmerie and Scott, 2018; Wang et al., 2021; Vassiliou and Farias-Pereira, 2023). In addition, other studies have reported that high APOE expression suppresses ferroptosis through activation of the PI3K/AKT1 pathway and promotes macrophage polarization toward an M2 phenotype in PTC tumors (Li et al., 2025). Although macrophages shift toward an M2 phenotype, defective lipid efflux causes these cells to become lipid-laden macrophages. This leads to the emergence of pathogenic M2 macrophages: cells that continue to express M2-associated genes but lose essential functional capacities. Lipid-laden macrophages display a unique metabolic profile distinct from classical M1 and M2 macrophages. During efferocytosis, these macrophages accumulate lipids within lysosomes, which are difficult to degrade, leading to lysosomal stress. To compensate, cells activate the MiT–TFE pathway to induce lysosomal biogenesis and autophagy. However, the excessive lipid load causes autophagy failure. Accumulation of p62 occurs as a consequence of impaired autophagy, further contributing to immune suppression. Meanwhile, PPARγ/LXR signaling, normally activated during efferocytosis, remains active but becomes skewed toward suppressing inflammation (inhibiting M1 phenotype) rather than restoring metabolic balance. This results in an immunosuppressive yet dysfunctional immune phenotype (Van Eijk and Aerts, 2021; Schilperoort et al., 2023). Defective efferocytosis leads to the accumulation of cellular debris, which is recognized as DAMPs, triggering chronic inflammation, fibrosis, and tissue necrosis. M2-like lipid-laden macrophages continue to express tissue-repair mediators; however, under abnormal metabolic conditions, their effects become maladaptive, promoting pro-fibrotic and pro-thrombotic activity. TGF-β increases due to PPARγ/LXR activation and false resolution signals from partial efferocytosis. Elevated TGF-β induces fibroblast activation, collagen synthesis, and progressive fibrosis in organs such as the liver, spleen, kidneys, and heart (Yang et al., 2023). Additionally, interactions between M2-like lipid-laden macrophages and fibrin or coagulation factors such as thrombin via PAR-1 activation enhance the expression of TGF-β, VEGF, and PAI-1, sustaining a pro-fibrotic and pro-thrombotic microenvironment. Consequently, coagulation is no longer protective but becomes a driver of chronic inflammation and tissue thrombosis (Koncz et al., 2023). The combination of these failures may contribute to the development of multi-organ dysfunction, thereby increasing mortality risk in IEM.

This study revealed that APOE is a principal gene exhibiting significant differential expression and strong associations with pathways related to lipid handling, immune modulation, and cellular stress response (Khalil et al., 2021; Van Eijk and Aerts, 2021; Theobald et al., 2024; Zandl-Lang, 2024; Ferreira et al., 2025). In the setting of IEM, chronic lipid accumulation gives rise to a maladaptive phenotype known as pathogenic M2 lipid-laden macrophages (Remmerie and Scott, 2018; van Eijk and Aerts, 2021; Wang et al., 2021; Vassiliou and Farias-Pereira, 2023; Ferreira et al., 2025). These cells maintain the expression of reparative mediators but lose critical homeostatic functions, including efferocytosis, resulting in an immunosuppressive and dysfunctional phenotype (Van Eijk and Aerts, 2021; Schilperoort et al., 2023) This maladaptive state triggers profibrotic and prothrombotic signaling pathways, contributing to persistent inflammation, fibrosis, and coagulation abnormalities (Koncz et al., 2023; Yang et al., 2023). The sustained presence and activity of these macrophages promote chronic tissue injury and multi-organ dysfunction, ultimately increasing mortality risk in patients with IEM (van Eijk and Aerts, 2021; Koncz et al., 2023; Schilperoort et al., 2023; Yang et al., 2023). These findings were further supported by morphological examination of bone marrow smears and cerebrospinal fluid, which demonstrated an overexpression of the lipid-laden macrophage phenotype. The convergence of bioinformatics prioritization and cytomorphological observations generates the hypothesis that APOE-related lipid pathways may be relevant to macrophage lipid-handling abnormalities in selected IEM contexts. However, the two evidence streams remain indirect: the bioinformatics analyses were not patient-specific, and the clinical cases were not evaluated for APOE genotype, expression, or protein abundance. The present findings therefore do not establish that APOE caused the observed macrophage phenotype.

Beyond its role in metabolic disease, the findings of this study may have broader implications for host immune responses in conditions involving intracellular pathogens. Macrophages serve as primary host cells for a wide range of intracellular bacteria, and their functional state critically determines the balance between pathogen clearance and persistence (Kaufmann, 2001; Flannagan et al., 2009). The lipid-laden, functionally impaired macrophage phenotype identified in this study may represent a metabolically reprogrammed state with reduced antimicrobial capacity, including impaired phagolysosomal function, altered autophagy, and diminished oxidative responses (Kaufmann, 2001; Russell et al., 2010). Such alterations could create a permissive intracellular environment that favors pathogen survival and persistence.

In this context, APOE-associated lipid dysregulation may contribute to a host microenvironment that is less effective in controlling intracellular infections. Lipid accumulation within macrophages can be exploited by intracellular pathogens as a nutrient source or as a strategy to evade immune responses (Russell et al., 2010; Cumming et al., 2018). For example, lipid-rich foamy macrophages are a well-recognized niche that supports the persistence of *Mycobacterium tuberculosis* within host tissues (Peyron et al., 2008; Russell et al., 2010). These observations suggest that metabolic conditions such as IEM may indirectly influence infection susceptibility and treatment outcomes through host immune reprogramming. Therefore, the APOE–macrophage axis identified in this study should be viewed as a potential candidate pathway for future investigation into host-directed therapeutic strategies aimed at restoring macrophage function and improving intracellular pathogen clearance (Zumla et al., 2016).

This study has several limitations. First, its observational and hypothesis-generating design does not permit causal inference regarding the relationships among APOE, lipid dysregulation, macrophage activation, and disease progression. The clinical component was also limited to two genetically related twin patients from a single family. These cases cannot be considered fully independent observations and may not represent the substantial genetic and phenotypic heterogeneity of IEM. Accordingly, the clinical findings should not be generalized to the broader IEM population. A further limitation is that the twin cases lacked definitive molecular and biochemical classification. Patient-specific genetic testing, including APOE genotyping or broader sequencing, was not available, and the specific metabolic pathway underlying disease progression could not be established. The observed clinical and cytomorphological phenotypes therefore cannot be directly attributed to APOE-associated mechanisms. The proposed APOE–macrophage relationship should instead be interpreted as a hypothesis derived from integrative bioinformatics analyses and considered alongside descriptive clinical observations, rather than as a disease-specific or experimentally established mechanism.

The pathological findings were primarily based on cytomorphological assessment of bone marrow and cerebrospinal fluid specimens. Although vacuolated, lipid-laden macrophage morphology was observed, macrophage immunophenotyping, lipid-specific characterization, APOE transcript or protein measurement, and APOE immunostaining were not performed. Consequently, the macrophage polarization state, functional activity, and tissue-level involvement of APOE could not be directly determined. No functional perturbation experiments were conducted to examine whether altered APOE expression or activity affects macrophage lipid handling, lysosomal function, autophagy, inflammatory signaling, or cell polarization. Similarly, several downstream processes included in the proposed model, such as intracellular pathogen persistence, ferroptosis, fibrosis, thrombosis, and multi-organ dysfunction, were not directly evaluated. These processes are therefore presented as literature-supported and biologically plausible hypotheses rather than experimentally demonstrated pathways in the present study. Finally, the study did not include an independent clinical validation cohort. Although the bioinformatics findings were examined across multiple annotation resources and single-cell reference datasets, such cross-database convergence does not constitute replication in an independent patient population. Future studies should validate these findings in larger cohorts of unrelated, genetically and biochemically characterized patients and incorporate patient-specific sequencing, comprehensive metabolic profiling, APOE expression and protein analyses, macrophage immunophenotyping, and functional experiments.

## 5 Conclusion

This integrative analysis prioritizes APOE as a gene associated with lipid metabolism pathways and macrophage-related reference signatures in IEM. The clinical cases independently demonstrated macrophage activation and vacuolated macrophage morphology consistent with intracellular lipid accumulation; however, they did not provide patient-specific evidence linking APOE to these findings or establish an M2 polarization state. The convergence of the bioinformatics and clinical observations supports the hypothesis that altered lipid-handling pathways may be associated with macrophage dysfunction in specific IEM contexts. This relationship remains associative and requires validation in molecularly characterized patient cohorts through macrophage immunophenotyping, APOE expression and protein analyses, and functional perturbation studies. Accordingly, the APOE–macrophage relationship should be regarded as a hypothesis for further investigation rather than an established disease mechanism or therapeutic target.

## Data Availability

The original contributions presented in the study are included in the article/Supplementary Material, further inquiries can be directed to the corresponding author/s.
